# Polystyrene Microplastics Postpone APAP-Induced Liver Injury through Impeding Macrophage Polarization

**DOI:** 10.3390/toxics10120792

**Published:** 2022-12-16

**Authors:** Jing Liu, Lecong Zhang, Fang Xu, Songyan Meng, Haitian Li, Yang Song

**Affiliations:** 1 College of Eco-Environmental Engineering, Guizhou Minzu University, Guiyang 550025, China; 2 The Institute of Karst Wetland Ecology, Guizhou Minzu University, Guiyang 550025, China; 3 State Key Laboratory of Environmental Chemistry and Ecotoxicology, Research Center for Eco-Environmental Sciences, Chinese Academy of Sciences, Beijing 100085, China

**Keywords:** polystyrene microplastics, acetaminophen, inflammation, macrophage, polarization

## Abstract

Polystyrene microplastics (PS MPs) are micrometer-scale items degraded from plastics and have been detected in various organisms. PS MPs have been identified as causing cognitive, cardiac, intestinal, and hepatic damage. However, their role in liver regeneration under drug-induced liver injury remains unknown. Thus, the current study aims to evaluate the impact of PS MPs on liver repair during APAP hepatotoxicity. PS MPs pretreatment exacerbates mice mortality and hepatocyte apoptosis, suppresses hepatic cell proliferation, and disturbs the inflammatory response in the APAP-induced damage model. Further mechanism exploration uncovers that prior PS MPs administration is sufficient to recruit neutrophils and macrophages, which are necessary for tissue recovery in the acute liver injury model. However, the polarization capacity of macrophages to anti-inflammatory sub-type is significantly delayed in PS MPs plus APAP group compared to the single APAP group, which is the leading cause of tissue repair suppression. Overall, the current study supports a new insight to realize the toxicity of PS MPs in acute liver injury, which should be considered in health risk assessment.

## 1. Introduction

Microplastics (MPs) are ubiquitous environmental pollutants characterized as particles with diameters from 0.1 μm to 5 mm. Traceability investigation indicates MPs are mainly derived from textile, medicine, and personal care products, while additional hydrolysis, photochemistry, and biodegradation promote the production of smaller items [1]. Deep research on humans identifies that MPs exist in the placenta [2], sputa [3], stool [4], and lung [5]. Especially, about 39,000–52,000 particles have been estimated to be consumed annually [6]. Polystyrene (PS) MPs rank top three in all types of plastics, confirming that PS MPs are the top three fragments in aqueous samples [7]. These investigations indicated the importance of evaluation of PS MPs toxicity. A mounting body of studies confirms that MPs are a health risk pollutant to cause cognitive impairment, cardiac damage, and intestinal injury [8]. Due to the pivotal role of the liver in detoxification, it frequently suffers from excessive attacks. PS MPs can induce oxidative stress, inflammatory response, metabolism disorder, apoptosis, pyroptosis, and ferroptosis in the liver [9,10,11,12]. Further co-exposure of MPs with epoxiconazole or cyclophosphamide exhibits excessive oxidative stress, more inflammatory cell infiltration, and increased serum alanine transaminase (ALT) [13,14]. However, whether existing PS MPs in the liver would impact hepatic recovery under acute liver injury is still uncertain. Hence, the current study aims to evaluate the role of PS MPs in drug-induced liver injury.

Acetaminophen (APAP) is a notorious compound to induce acute liver injury [15]. Actually, it is the main component of drugs used for pain and fever in limited doses. However, excessive APAP uptake is a leading cause of acute liver injury in the United States and Europe [16,17]. Upon APAP intake, it will metabolize to N-acetyl-p-benzoquinone imine (NAPQI) mediated by cytochrome P450 2E1 (CYP2E1), which is accompanied by glutathione depletion, mitochondrial dysfunction, and hepatocytes necrosis [15,18]. Hepatocyte necrosis releases damage-associated molecular patterns (DAMPs), further triggering Kupffer cells (resident macrophages in the liver) to produce pro-inflammatory cytokines, such as TNF-α and IL-6 [19]. These pro-inflammatory cytokines provoke infiltration and the recruitment of macrophages and neutrophils to the injury area to remove debris and initiate recovery [20,21]. However, the achievement of liver regeneration depends on the state of macrophages, including Kupffer cells and monocyte-derived macrophages [21,22]. In the early stage of liver injury, pro-inflammatory macrophages (M1 type) dominate the liver. In contrast, anti-inflammatory macrophages (M2 type) are occupied in the late recovery stage [23,24]. M1 type macrophages are activated by IFN-γ, which produces pro-inflammatory factors, whereas IL-4 induces oppositely M2-type macrophages to release anti-inflammatory factors to promote remodeling in the damaged area [25,26]. Significantly, during the regeneration of APAP hepatotoxicity, the conversion of M1/M2 macrophages relies on neutrophil-produced reactive oxygen species (ROS) [27]. Although the inflammatory response induced by MPs has been evaluated, how it works under a complex liver repair process is still incompletely understood.

Thus, the current study intends to uncover the role of PS MPs in APAP-induced acute liver injury, then explore the possible mechanism. In conclusion, our findings confirmed that PS MPs exposure aggravates APAP-induced liver injury by hindering the conversion of macrophages.

## 2. Materials and Methods

### 2.1. Mice

Male Balb/C mice (7 weeks of age) were purchased from Changsha Tianqin Biotechnology Company (Changsha, China). The animal study was approved by Guizhou Minzu university and followed the NIH’s guidelines. After adaptive feeding for one week, the mice were randomly divided into 6 mice per group, including control (ddH_2_O only), APAP (Sigma-Aldrich, St. Louis, MO, USA), PS MPs (characterized in Figure S1, Tianjin Baseline ChromTech Research Centre, Tianjin, China), and APAP combined with PS MPs groups. The whole experiment was sustained for 9 days. PS MPs were suspended in ddH_2_O. APAP was dissolved in ethanol, then diluted in ddH_2_O. PS MPs exposure mice were pretreated with 10 mg/kg body weight PS MPs for successive 7 days through intragastric administration. During this time, the other groups received ddH_2_O only. After 7 days, the APAP and APAP with PS MPs groups received a single intraperitoneal injection of 300 mg/kg APAP. After an additional 2 days, mice were sacrificed, and liver sections were stored at −80 °C. Collected blood was centrifuged at 800 g for 10 min, and the supernatant was gathered and stored for further studies.

### 2.2. Serum ALT Assay

Serum ALT activity in each sample was determined using an ALT assay kit (Nanjing Jiancheng Bioengineering Institute, Nanjing, China) following the manufacturer’s instructions. The absorbance of each sample was determined using an automatic microplate reader at 510 nm (Synergy H1, BioTek, Winooski, VT, USA).

### 2.3. Histochemical Staining

The mouse liver section was fixed in 4% paraformaldehyde for 48 h, then encapsulated into wax and stained using a hematoxylin and eosin (H&E) staining kit (Solarbio, Beijing, China) following the manufacturer’s procedure. To remove paraffin, the slides were incubated with xylene and different concentrations of alcohol solutions. After being washed with water, slides were stained by hematoxylin for 12 min and eosin aqueous for 2 min. Following quick water washing, slides were dehydrated in different proportions of alcohol solutions. Finally, the slides were made transparent using xylene and sealed with resinene. The pictures were captured by CX33 (Olympus, Tokyo, Japan).

### 2.4. Terminal Deoxynucleotidyl Transferase dUTP Nick end Labeling (TUNEL) Staining

The TUNEL apoptosis detection kit used in the current study was purchased from KeyGEN Biotech (Nanjing, China). The experimental procedure was performed following the manufacturer’s description. Paraffin-embedded liver sections were incubated in xylene and alcohol with different concentrations. Then, slides were rinsed three times in PBS and further incubated with Proteinase K working solution. The slides interacted with 3% H_2_O_2_ at room temperature, then re-incubated with terminal deoxynucleotidyl transferase (TDT) enzyme, streptavidin-horseradish peroxidase (HRP) solution, and 3,3′-diaminobenzidine (DAB) working solution, respectively. Additionally, slides were re-dyed with hematoxylin solution. Following dehydration in alcohol with different proportions, and obtaining transparency using xylene, the slides were sealed with resinene. Images of staining slides were captured by CX33.

### 2.5. Immunohistochemistry (IHC)

After being fixed with 4% paraformaldehyde, liver specimens were incubated with 1% goat serum (ZSGB-BIO, Beijing, China) for 1 h at room temperature in a humidified chamber. Then, the slides were stained with primary antibodies for 4 h. Primary antibodies included Ki67 (dilution: 1:500, Abcam, Cambridge, UK) and Ly6G (dilution: 1:500, Abcam, Cambridge, UK). After 3 times washing by PBS, the liver sections were further covered by HPR decorated goat anti-mouse or anti-rabbit IgG (ZSGB-BIO, Beijing, China) for 1 h. After rinsing slides 3 times with PBS, the sections were further enhanced through DAB substrate solution for 30 min. Finally, slides were counterstained with hematoxylin (Solarbio, Beijing, China) for 1 min. Images were captured by CX33.

### 2.6. RNA Extraction and Real-Time Quantitative PCR (RT-qPCR) Analysis

According to the manufacturer’s instructions, total RNA was isolated from the liver section by Trizol reagent (Invitrogen, Carlsbad, CA, USA). cDNA was synthesized following a reverse transcription kit as the protocol described (Roche, Hilden, Germany). The expressions of relative target genes were evaluated via SYBR^®^ green qPCR mix (Promega, Madison, WI, USA) on Roche LightCyler96 (Roche, Basel, Switzerland). Hypoxanthine-guanine phosphoribosyl transferase (HPRT) was used as a housekeeping gene to normalize relative gene expressions. The primer sequences used are presented in Table S1.

### 2.7. Protein Isolation and Western Blotting Analysis

Grinded liver homogenate was collected. Then, protein was extracted using radio-immunoprecipitation assay (RIPA) lysis buffer (Solarbio, Beijing, China) containing a protease inhibitor cocktail from Roche Life Science. The protein concentration was determined by bicinchoninic acid (BCA) protein assay kit (Solarbio). Subsequently, equal amounts of proteins for each sample were subjected to 8% SDS-PAGE, and the separated proteins were transferred to nitrocellulose membranes. After 2 h blocking by 5% nonfat milk at room temperature, the membranes were further incubated with primary antibody β-actin (1:2000, Proteintech, Wuhan, China) and CD68 (1:1000, Proteintech) at 4 °C overnight. Next, the membranes were washed 3 times using Tris-buffered saline with Tween 20 (TBST), then incubated with HPR labeled goat anti-mouse and anti-rabbit secondary antibody (1:2000, Proteintech) for an additional 1 h. Finally, the membranes were visualized using SuperSignal^TM^ West Pico PLUS (Thermo Fisher, Waltham, MA, USA).

### 2.8. Statistical Analysis

All data are presented as mean ± standard deviation (SD). SPSS statistics 23 software (IBM Corp., Armunk, NY, USA) was chosen for data analysis via one-way ANOVA. A *p* value less than 0.05 was considered significant. 

## 3. Results and Discussion

### 3.1. PS MPs Promote APAP-Induced Liver Damage

**Figure 1:**
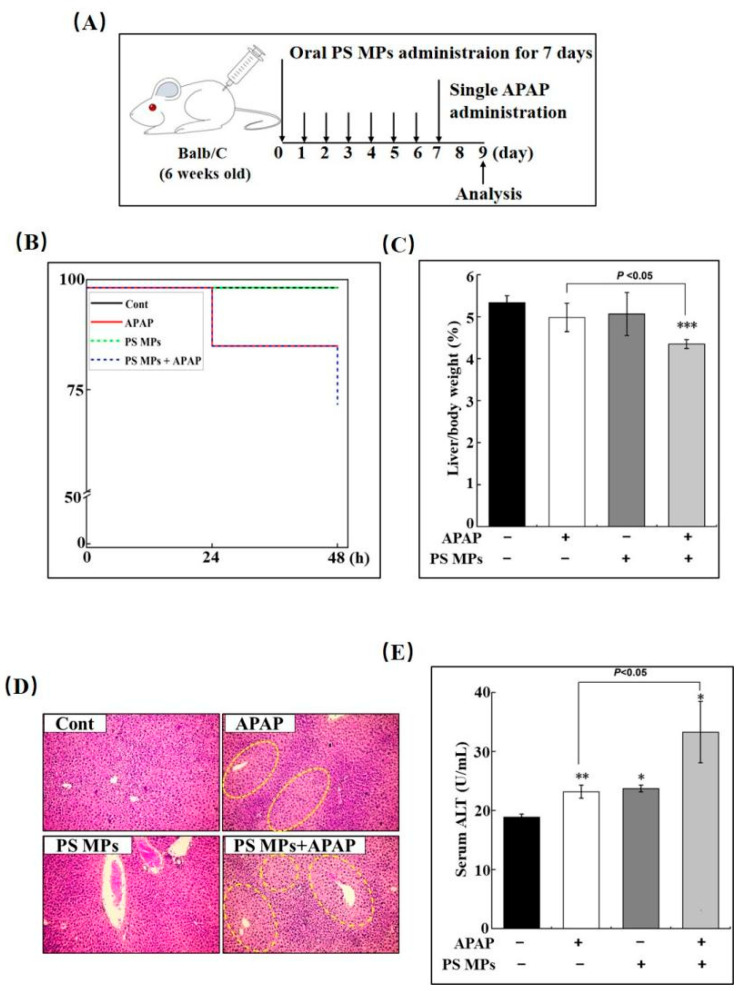
PS MPs treatment exacerbates APAP-caused liver injury. (**A**) A schematic diagram of the experiment design. (**B**) Mortality and (**C**) liver and body weight ratio of mice received APAP, PS MPs and APAP combined with PS MPs (*n* ≥ 3). (**D**) The representative histological changes of liver section upon APAP, PS MPs and APAP combined with PS MPs through intragastric administration via H&E staining (Original magnification was 100×. Damaged area was labeled inside yellow circle). (**E**) The activity of ALT in sera of mice. Asterisk (*) indicates *p* < 0.05, ** denotes *p* < 0.01, *** denotes *p* < 0.001 compared to the control group.

To assess the role of PS MPs in APAP-induced liver damage and recovery, we pretreated mice daily with 10 mg/kg bodyweight PS MPs for seven days to quickly achieve MPs accumulation in the liver based on a previous study [28]. Then, we administrated mice with 300 mg/kg APAP to construct a liver damage model (Figure 1A). Upon APAP administration, the liver will turn to the resolution phase (48–72 h) after a short, distinct injury (0–24 h) [29]. Hence, 48 h after APAP treatment was chosen as the time point to evaluate the regenerative capacity of PS MPs under APAP-induced hepatotoxicity in the current study. As indicated in Figure 1B, one mouse died in a single APAP group and APAP combined with PS MPs group within 24 h after APAP administration. Another mouse died in APAP combined with PS MPs group between 24 and 48 h, which indicates that prior PS MPs exposure aggravated APAP-caused damage. Then, the mice liver and body weight were monitored. As shown in Figure 1C, the liver-to-body weight ratio is decreased in APAP and PS MPs groups, especially in APAP combined with PS MPs group (*p* < 0.001). In contrast to APAP group, APAP plus PS MPs group has a more severe liver/body weight ratio reduction (Figure 1C, *p* < 0.05). To further estimate liver toxicity, H&E staining was conducted. In Figure 1D, the liver changes pale, and a significant decrease in cell number (indicated in the yellow circle) can be observed in the APAP group. However, this became more severe in APAP with PS MPs group. ALT is a key marker to indicate the function of the liver. Compared to the control group, serum ALT in APAP, PS MPs, and APAP with PS MPs groups increased 21% (*p* < 0.01), 23% (*p* < 0.05), and 73% (*p* < 0.05), individually (Figure 1E). Compared to APAP group, serum ALT in APAP and PS MPs co-exposure group increased 43% (*p* < 0.05), indicating heavier damage. The synergistic adverse effect on liver damage was also observed in 70 nm silica particle-APAP and PS NPs-APAP co-exposure models [30,31]. However, the studies concluded that gold NPs and silver NPs could reverse the ALT and anti-oxidative enzymes by harnessing reactive oxygen species after 2 g/kg APAP oral administration [32,33]. These differences might contribute to the dosage of APAP and the different types and construction of materials.

### 3.2. PS MPs Pretreatment Impacts Apoptosis and Proliferation

**Figure 2:**
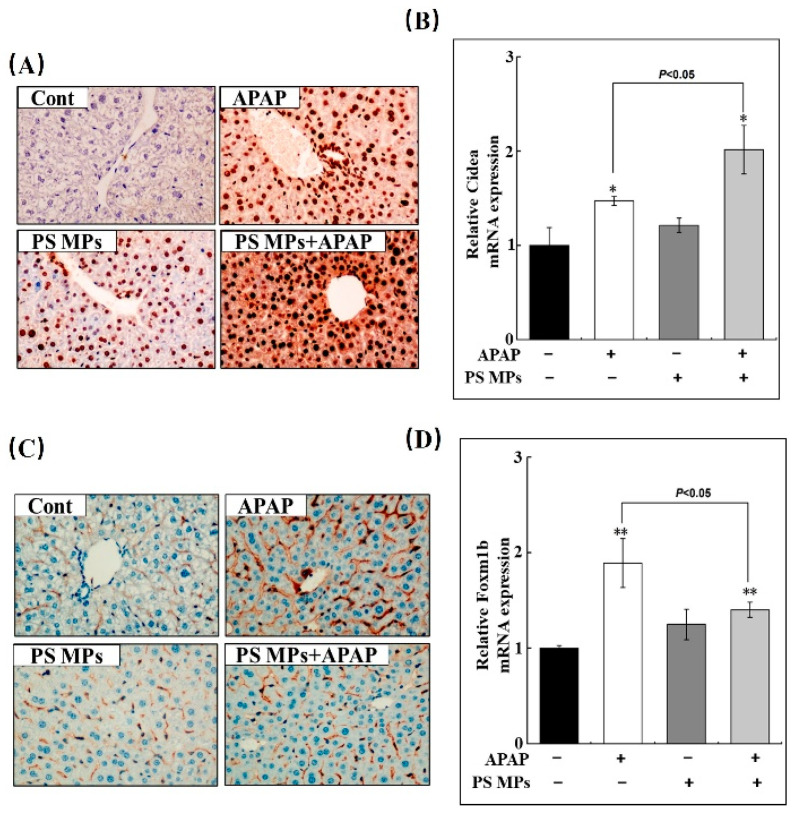
PS MPs accelerate cell apoptosis and suppress cell proliferation in APPA-induced liver damage. (**A**) Representative images of cell apoptosis in the liver section of mice after APAP, PS MPs and APAP combined with PS MPs administration through TUNEL staining (Brow dot, 400×). (**B**) The variation of hepatic Cidea mRNA in mice (*n* ≥ 3). (**C**) Immunohistochemical staining of Ki67 in liver section, positive cells were presented in brown (400×). (**D**) The changes of hepatic Foxm1b level, which indicates cell proliferation after APAP, PS MPs and APAP combined with PS MPs exposure (*n* ≥ 3). *: *p* < 0.05, **: *p* < 0.01 compared to control.

The prognosis of APAP hepatotoxicity depends on the balance between cell death and regeneration, while the latter exhibits cell proliferation requirements [34]. However, necrosis was considered the reason for hepatotoxicity in an early study, and further mechanism research confirmed the involvement of apoptosis [15]. Thus, we used TUNEL staining to detect cellular apoptosis in the liver. APAP, PS MPs, and APAP combined with PS MPs groups all induced different degrees of cell apoptosis in the liver (brown dots), especially in APAP with PS MPs group (Figure 2A). Cidea, the gene that participates in cell death, was chosen to evaluate cell apoptosis in the current study [35]. Compared to the control group, Cidea mRNA expression was increased 1.5-fold and 2-fold in APAP and APAP with PS MPs groups, respectively (Figure 2B, *p* < 0.05). Compared to APAP administrated group, Cidea transcriptional expression increased 33% in APAP combined with PS MPs group (Figure 2B, *p* < 0.05). Subsequently, Ki67, a protein necessary for DNA replication [36], was detected through IHC. Compared to control group, Ki67 expression is significantly increased in APAP, PS MPs, and APAP combined PS MPs groups (Figure 2C, brown dots). Still, the expression of Ki67 in APAP with the PS MPs group is obviously less than in the APAP group, indicating that cell proliferation capacity decreased in the APAP combined PS MPs group. Further supporting these results, Forkhead Box M1b (Foxm1b), the gene that participates in cell proliferation [34], was further examined. As shown in Figure 2D, hepatic Foxm1b increased 1.8- and 1.4-fold in APAP and APAP with PS MPs groups, respectively (*p* < 0.01), but the hepatic Foxm1b mRNA level in the APAP group is much higher than APAP with PS MPs group (Figure 2D, *p* < 0.05). These results indicate APAP plus PS MPs exposure not only worsens cell damage, but also weakens cell proliferation capacity. A similar role of PS MPs in cell apoptosis induction and cell proliferation inhibition is commonly observed [12,37,38].

### 3.3. PS MPs Pretreatment Impacts the Inflammatory Response

**Figure 3:**
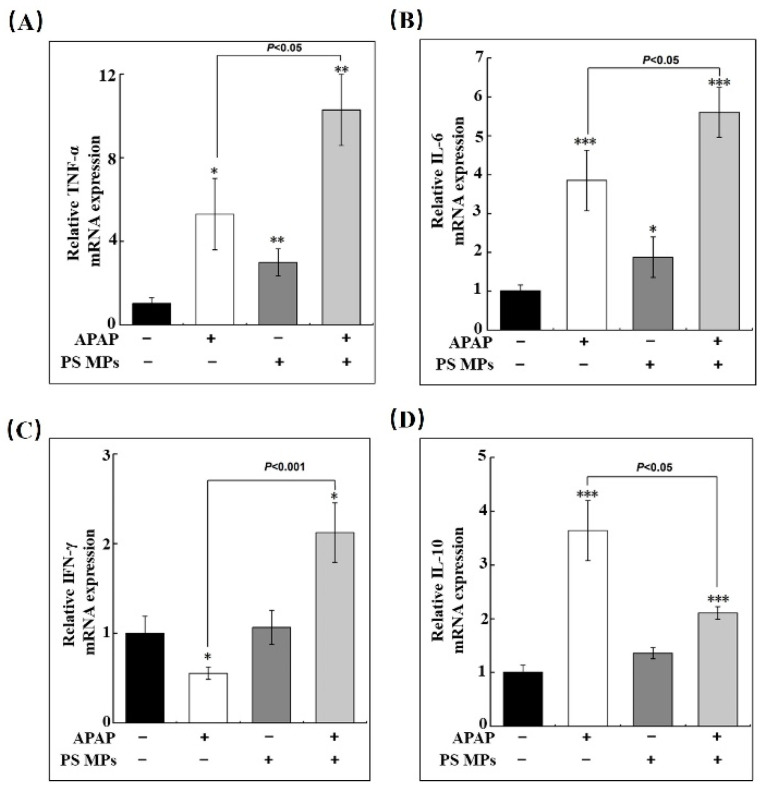
PS MPs pretreatment enhances APAP-caused inflammatory response. The variation of transcriptional levels of (**A**) TNF-α, (**B**) IL-6, (**C**) IFN-γ, and (**D**) IL-10. (*n* ≥ 3). *: *p* < 0.05, **: *p* < 0.01, ***: *p* < 0.001 compared to control.

APAP-caused DAMPs are the activators of the inflammatory response, which initiates the production of inflammatory cytokines associated with the type of immune cells and impacts the APAP-induced damage and regeneration [39]. Hence, we detected tumor necrosis factor-alpha (TNF-α), interleukin-6 (IL-6), and interferon-gamma (IFN-γ), which belong to the pro-inflammatory cytokines family. Compared to the control group, TNF-α mRNA expressions in APAP, PS MPs, and APAP combined PS MPs groups increased 5.5- (*p* < 0.05), 3.2- (*p* < 0.01), and 10-fold, respectively (*p* < 0.01) (Figure 3A). The variation of TNF-α mRNA in the APAP with PS MPs group is more potent than the APAP group, indicating that APAP with PS MPs exhibited a severe pro-inflammatory response (Figure 3A, *p* < 0.05). Similar to TNF-α transcriptional variation, IL-6 level was increased 3.8- (*p* < 0.001), 1.9- (*p* < 0.05), and 5.5-fold (*p* < 0.001) upon APAP, PS MPs, and APAP with PS MPs administration, Figure 3B. Furthermore, IFN-γ mRNA expression displayed ~50% reduction in the APAP administration group (*p* < 0.05), and a slight increase was found in the PS MPs group. However, IFN-γ mRNA expression increased 2.1-fold in APAP combined PS MPs administration group compared with the control group (*p* < 0.05) (Figure 3C). Interleukin-10 (IL-10) is classified as an anti-inflammatory factor to inhibit pro-inflammatory cytokines from achieving tissue regeneration [40]. In contrast to TNF-α, IL-6, and IFN-γ, although APAP and APAP with PS MPs groups enhanced IL-10 mRNA expressions (*p* < 0.001), the variation of APAP with PS MPs group is significantly lower than APAP group (Figure 3D, *p* < 0.05). The mRNA variation of anti-inflammatory and pro-inflammatory genes indicates that APAP exhibits apparent anti-inflammation potency rather than APAP plus PS MPs. The above variation tendency of inflammatory cytokines is in accord with APAP [41]. Still, a recent study concerning microplastics observed similar pro-inflammatory cytokines in the liver [42]. Our results are also consistent with the research of PS MPs on acute colitis to induce inflammatory effects [43]. To sum up, mice co-exposed with APAP and PS MPs exhibited severe pro-inflammatory impacts rather than an anti-inflammatory state. We speculated that the initiation of liver recovery is impeded.

### 3.4. PS MPs Pretreatment Facilitates Neutrophils Recruitment

**Figure 4:**
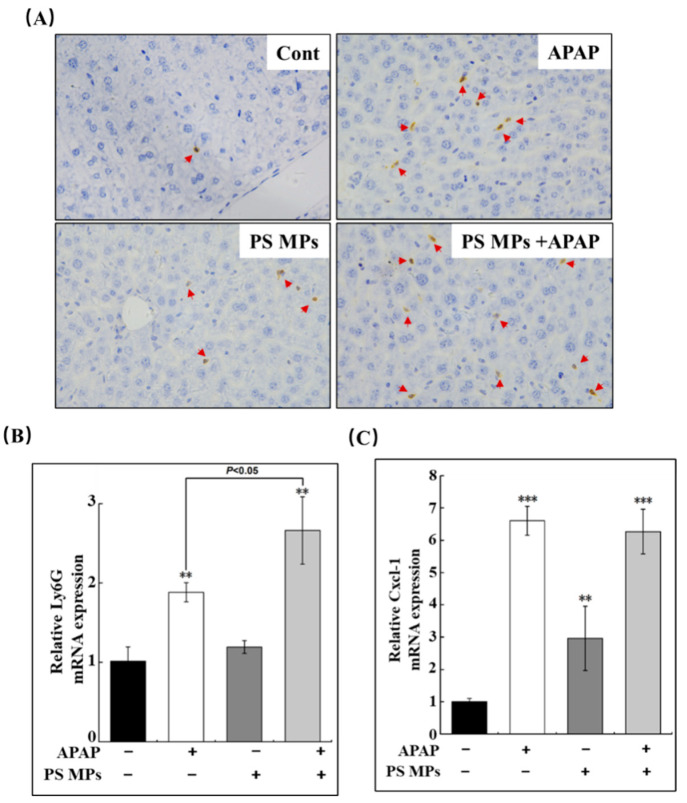
PS MPs pretreatment increased neutrophil recruitment capacity in mice after APAP injury. (**A**) Representative immunohistochemical staining of liver biopsy with Ly6G. Ly6G positive cells appeared in brown dots (red arrows). The changes of (**B**) Ly6G and (**C**) Cxcl-1 mRNA upon APAP, PS MPs and combined exposure (*n* ≥ 3). **: *p* < 0.01, ***: *p* < 0.001 compared to control.

Due to the pro-inflammatory state being dominated in APAP combined with the PS MPs group in the liver, the recruitment of neutrophils should be considered. Neutrophil has been identified to participate in liver recovery by boosting macrophage conversion from the pro-inflammatory to the anti-inflammatory stage [27,29]. Next, we tested the neutrophil marker Ly6G in the liver after APAP treatment for 48 h. As shown in Figure 4A, APAP and APAP-combined with PS MPs administration group had conspicuous Ly6G in the liver section (brown dots, as indicated by red arrows), but APAP with PS MPs displayed more capability in neutrophils recruitment than the APAP group. The mRNA variation was also tested to confirm the expression of Ly6G. According to the Ly6G IHC result, hepatic Ly6G mRNA expressions were increased in APAP and APAP with PS MPs groups compared to the control (Figure 4B, *p* < 0.01). The relative Ly6G mRNA level in APAP with PS MPs group was higher than APAP group (Figure 4B, *p* < 0.05). Chemokine C-X-C motif ligand 1 (Cxcl-1) was considered a chemoattractant for neutrophil infiltration [44]. Thus, we evaluated Cxcl-1 transcriptional expression in the liver. In Figure 4C, Cxcl-1 mRNA levels were boosted 6.6-, 3-, and 6.2-fold within APAP, PS MPs, and APAP plus PS MPs administrated groups, respectively, while there is no disparity between APAP and APAP plus PS MPs groups (Figure 4C). The changes of Cxcl-1 expression on APAP administration are in line with a previous study [45]. Thus, the above information declared that APAP and APAP with PS MPs groups could accelerate neutrophil recruitment, while APAP with PS MPs co-treatment exhibits more robust capacity than APAP. Thus, the delayed liver recovery activation could not be attributed to neutrophil recruitment in the current study.

### 3.5. PS MPs Pretreatment Promotes Macrophage Recruitment

**Figure 5:**
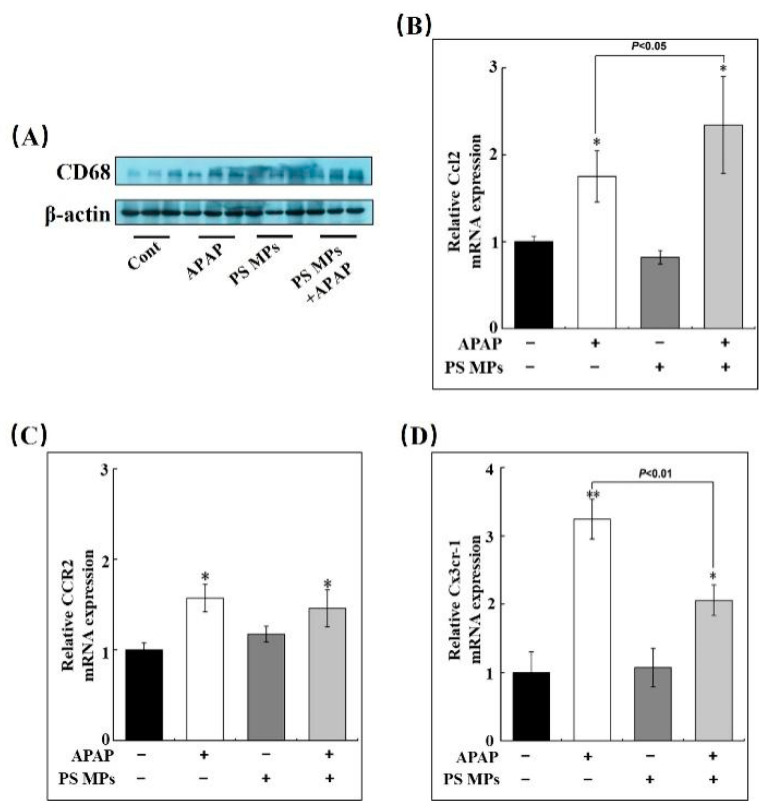
PS MPs pretreatment exhibited strong macrophage recruitment capacity after APAP injury. (**A**) The changes in CD68 expression were analyzed by Western blotting. Relative (**B**) Ccl2, (**C**) CCR2 and (**D**) Cx3cr-1 mRNA expression (*n* ≥ 3). *: *p* < 0.05, **: *p* < 0.01 compared to control.

Macrophages are under investigation due to their particular role in removing necrotic debris to achieve hepatic regeneration [26]. Thus, we checked their markers to assess accumulation capacity in the liver. The cluster of differentiation 68 (CD68) is defined as an indicator of resident macrophages [46], which was determined through Western blotting in the current study. In Figure 5A, CD 68 expression is higher in APAP, PS MPs, and APAP pretreated with PS MPs groups compared to the control (Figure 5A). Infiltrated monocyte-derived macrophages are non-negligible in tissue repair as well [24]. Ccl2 was responsible for monocyte-derived macrophage recruitment that was quantified through RT-qPCR. Significant Ccl2 expression was observed in the single APAP group and APAP co-exposure with PS MPs group (*p* < 0.05), but APAP combined with PS MPs group increased ~30% compared to the single APAP group (Figure 5B, *p* < 0.05). To confirm the infiltrated monocyte-derived macrophage in the liver, CC chemokine receptor 2 (CCR2), the marker of monocyte-derived macrophage, was considered [47]. As shown in Figure 5C, APAP and APAP plus PS MPs significantly up-regulated CCR2 mRNA transcriptional expression in the liver, respectively (*p* < 0.05), but no significance is observed between APAP and APPA plus MPs groups. Chemokine CX3C receptor 1 (Cx3cr1) is abundant in pro-resolving monocyte-derived macrophages rather than pro-inflammatory macrophages [27]. Subsequently, we checked Cx3cr1 expression in the liver section. Cx3cr-1 mRNA expression increased 3.3-fold in the APAP challenged group compared to the control (Figure 5D, *p* < 0.01). However, APAP combined PS MPs group was up-regulated by merely 2.1-fold (*p* < 0.05), which is lower than APAP treated group (Figure 5D, *p* < 0.01). Analogously, the alterations of Ccl-2, CCR2 and Cx3cr1 expression upon APAP are in line with the previous studies [48,49]. However, these results uncovered that APAP co-administrated with PS MPs is more capable of macrophage recruitment in the liver than APAP-treated mice. However, single APAP administration mice exhibited greater anti-inflammatory priority.

### 3.6. PS MPs Pretreatment Impedes Macrophage Conversion

**Figure 6:**
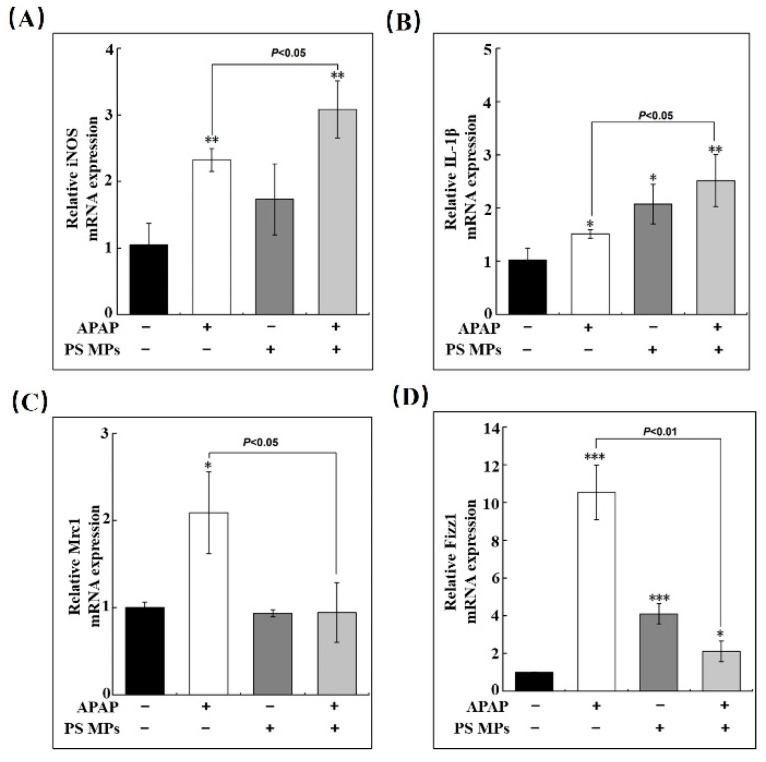
PS MPs treatment postponed macrophage transformation after APAP injury. The alterations of (**A**) iNOS, (**B**) IL-β, (**C**) Mrc1 and (**D**) Fizz1 in the liver after APAP, PS MPs and APAP combined with PS MPs administration (*n* ≥ 3). *: *p* < 0.05, **: *p* < 0.01, ***: *p* < 0.001 compared to cotrol.

In order to identify the states of macrophages in the liver which are associated with liver regeneration, further assessment of M1/M2 macrophage markers was performed since excessive M1-type macrophages would exacerbate liver damage [24]. As presented in Figure 6A, induced nitric oxide synthases (iNOS), a standard pro-macrophage marker, dramatically increased in APAP and APAP pretreated with PS MPs groups (2.3- and 3-fold respectively, *p* < 0.01) compared to control. Compared to the APAP group, APAP plus PS MPs group was further enhanced by 30% (Figure 6A, *p* < 0.05). Similar to iNOS, the variation of another -M1 gene, interleukin 1 beta (IL-1β), increased 1.5- (*p* < 0.05) and 2.4-fold (*p* < 0.01) upon APAP and APAP with PS MPs administrations (Figure 6B). Both iNOS and IL-1β results indicate that APAP and APAP with PS MPs treatments could enhance the transcriptional expression of pro-inflammatory factors. However, APAP with PS MPs treatment group has a more substantial impact than the APAP group. By contrast to iNOS and IL-1β, the expressions of M2 relative gene mannose receptor C-type 1 (Mrc1) and resistin-like molecule-alpha (Fizz1) are significantly increased in the APAP group, compared to the control group (*p* < 0.05). Surprisingly, no variation was found in APAP with PS MPs group (Figure 6C). In Figure 6D, Fizz1 mRNA is increased in all exposure groups compared to the control (*p* < 0.05). However, the APAP group showed approximately five-fold the Fizz1 mRNA expression of APAP plus PS MPs group (*p* < 0.01). The variations of representative genes of M1/M2 macrophages illustrate that APAP plus PS MPs prefer to perform at M1 type. Our results are consistent with the study of 500 nm MPs in C57BL/6J mice in that the M1 population was enhanced [50]. Collectively, we illustrated that macrophage polarization lagging is the leading cause of PS MPs-induced liver injury and repair.

## 4. Conclusions

In this study, we found APAP and PS MPs co-exposure induced severe liver damage, accompanied by delayed liver repair. Mechanistic research indicated that although the recruitment of macrophages and neutrophils is strengthened in the co-exposure group, the critical process to initiate M2-type macrophage conversion was significantly impeded. In summary, the current study proposed that drug-induced liver injury is enhanced with environmental pollutant exposure, which should be further considered in the risk assessment of human health.

## Supplementary Materials

The following supporting information can be downloaded at: https://www.mdpi.com/article/10.3390/toxics10120792/s1, Figure S1: Physico-chemical characterization of PS MPs; Table S1: Sequences of all gene primers.
