# Supplementary material for: Polystyrene Microplastics Postpone APAP-Induced Liver Injury through Impeding Macrophage Polarization
Source: Toxics. 2022 Dec 16;10(12):792. doi: 10.3390/toxics10120792 (PMC9781384; doi:10.3390/toxics10120792)
Supplement: Supplementary file 1 [file toxics-10-00792-s001.zip › toxics-2065815-supplementary.pdf]

Supporting Information

Polystyrene microplastics postpone APAP induced liver injury through impeding macrophage polarization

Jing Liu[1, 2, 3], Lecong Zhang[1], Fang Xu[1,3], Songyan Meng[1], Haitian Li[1], Yang Song[3,*]

1. College of Eco-Environmental Engineering, Guizhou Minzu University, Guiyang, 550025, China

2. The Institute of Karst Wetland Ecology, Guizhou Minzu University, Guiyang, 550025, China

3. State Key Laboratory of Environmental Chemistry and Ecotoxicology, Research Center for Eco-Environmental Sciences, Chinese Academy of Sciences, Beijing, 100085, China

*Corresponding Author: State Key Laboratory of Environmental Chemistry and Ecotoxicology, Research Center for Eco-Environmental Sciences, Chinese Academy of Sciences, 18 Shuangqing Rd, Haidian District, Beijing, 100085, China; E-mail: yangsong@rcees.ac.cn

Physicochemical property of PS MPs

To evaluate the impact of PS MPs on liver repair in APAP-induced liver injury model, the physicochemical property of the material was characterized. In Figure S1A, scanning electron microscopy (SEM) result indicates that MPs is round with a size of 300 nm. Further size distribution evaluation through dynamic light scattering (DLS) presented average size of MPs is approximately 320 nm (Figure 1B). From Figure 1C, the zeta potential of MPs is at low vacuum mode.

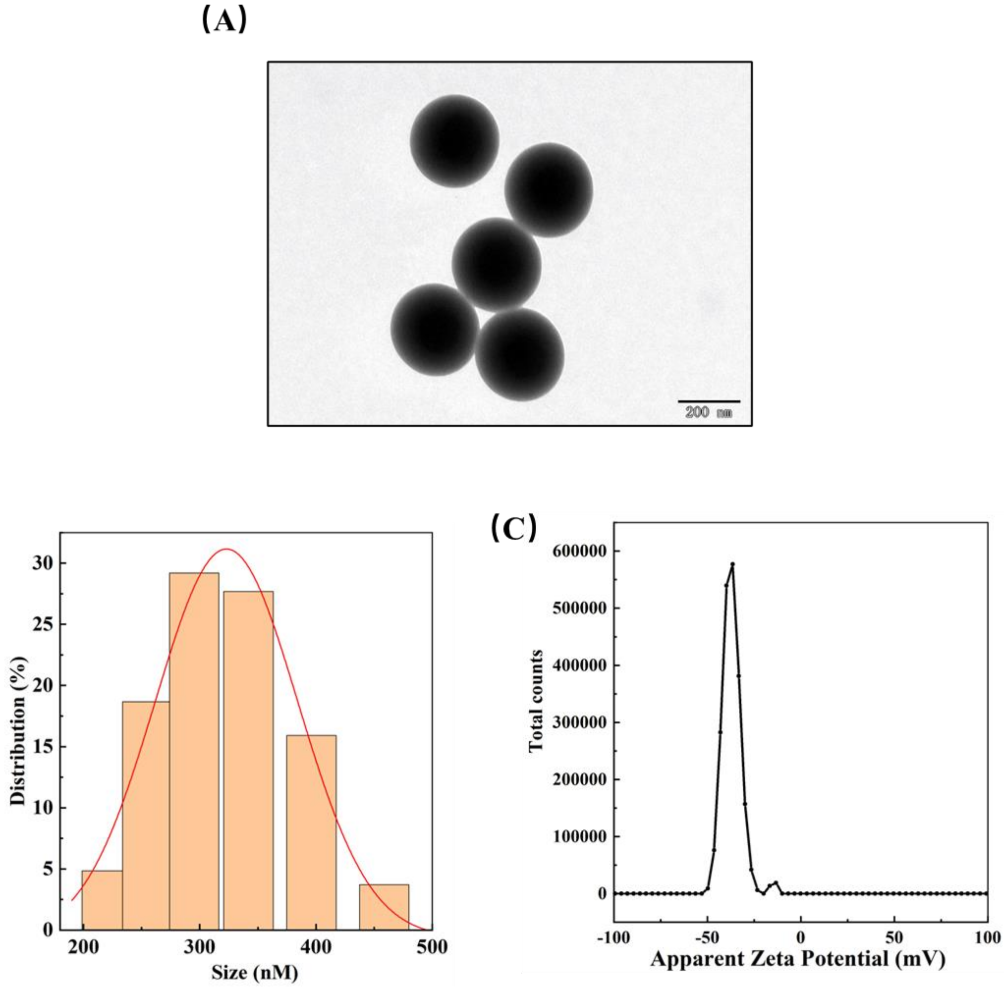

Figure S1 Physico-chemical characterization of PS MPs. (A) TEM images of PS MPs; (B) Hydrodynamic size and (C) zeta potential of PS MPs in distilled water.

Table S1. Sequences of all gene primers

S. No	Name	Primer sequences
1	Hprt	GCTTGCTGGTGAAAAGGACCTCTCGAAG<br>CCCTGAAGTACTCATTATAGTCAAGGGCAT
2	Cidea	CCTTTGGTGCTAGGCTTGG<br>TTCAAGGCCGTGTTAAGGA
3	Foxm1b	GTGTGCCTGTTCCCAAGC<br>CTGTTGTCCAGCGTGCAG
4	Tnf-α	CCCTCACACTCAGATCATCTTCT<br>GCTACGACGTGGGCTACAG
5	IL-6	CCAGAGCTGTGCAGATGAGT<br>CTGCAGCCACTGGTTCTGT
6	IFN-γ	CGGCACAGTCATTGAAAGCCTA<br>GTTGCTGATGGCCTGATTGTC
7	IL-10	GCTCTTACTGACTGGCATGAG<br>GCTCTTACTGACTGGCATGAG
8	Ly6G	GACTTCCTGCAACACAACTACC<br>ACAGCATTACCAGTGATCTCAGT
9	Cxcl-1	CTGGGATTCACCTCAAGAACATC<br>CAGGGTCAAGGCAAGCCTC
10	Ccl-2	AGGTCCCTATGGTGCCAATGT<br>CGGCAGGATTTTGAGGTCCA

11	CCR2	CCACATCTCGTT CTCGGTTTATC<br>CAGGGAGCACC GTAATCATAATC
12	Cx3cr-1	CAGCATCGACCGGTACCTT<br>GCTGCACTGTCCGGTTGTT
13	iNOS	TCTTTGACGCTCGGAACTGTAGCA<br>TAGGTCGATGCACAACTGGGTGAA
14	IL-1β	TGCCACCTTTTGACAGTGATG<br>AAGGTCCACGGGAAAGACAC
15	Mrc1	CTCTGTTCAGCTATTGGACGC<br>CGGAATTTCTGGGATTCAGCTTC
16	Fizz1	CCCTCCACTGTAACGAAGACTC<br>CACACCCAGTAGCAGTCATCC
